# Development and Validation of a Tool for the Prediction of Vancomycin-Resistant Enterococci Colonization Persistence—the PREVENT Score

**DOI:** 10.1128/Spectrum.00356-21

**Published:** 2021-09-15

**Authors:** Christian Boeing, Carlos L. Correa-Martinez, Franziska Schuler, Alexander Mellmann, André Karch, Stefanie Kampmeier

**Affiliations:** a Institute of Hygiene, University Hospital Münster, Münster, Germany; b Institute of Medical Microbiology, University Hospital Münster, Münster, Germany; c Institute of Epidemiology and Social Medicine, University of Münster, Münster, Germany; Houston Methodist Hospital

**Keywords:** contact precaution, prediction score, screening, vancomycin-resistant enterococci

## Abstract

Vancomycin-resistant enterococci (VRE) are nosocomial pathogens with increasing prevalence worldwide. Extensive hygiene measures have been established to prevent infection transmission in hospitals. Here, we developed a predictive score system (the *pre*dictive *v*ancomycin-resistant *ent*erococci [PREVENT] score) to identify the clearance or persistence in patients with a history of VRE carrier status at readmission. Over a cumulative 3-year period, patients with a positive VRE carrier status were included. The study population was recruited in two successive time periods and separated into training data for predictive score development and validation data for evaluation of the predictive power. The risk factors for persistent VRE colonization were analyzed in a univariable analysis before development of a logistic regression model based on the potential risk factors. The score points were determined proportionally to the beta coefficients of the logistic regression model. The data from 448 (79%) patients were used as the training data, and those from 119 (21%) as the validation data. Multivariable analysis revealed the following variables as independent risk factors: age of ≥60 years, hemato-oncological disease, cumulative antibiotic treatment for >4 weeks, and a VRE infection. The resulting logistic regression model exhibited an acceptable area under the curve (AUC) of 0.81 (95% confidence interval [CI], 0.72 to 0.91). The predictive score system had a sensitivity of 82% (95% CI, 65 to 93%) and a specificity of 77% (95% CI, 66 to 85%). The developed predictive score system is a useful tool to assess the VRE carrier status of patients with a history of VRE colonization. On the basis of this risk assessment, more focused and cost-effective infection control measures can be implemented.

**IMPORTANCE** Given the increasing relevance of VRE as nosocomial pathogens worldwide, infection prevention and control measures, including patient isolation and contact precautions, are indispensable to avoid their spread in the hospital setting. In this study, we developed and validated the PREVENT score, a tool for rapid risk assessment of VRE persistence in patients with a history of previous VRE colonization. The score is designed to be easily performed, employing clinical information available in a regular admission setting and immediately providing information to inform the decision of whether to adopt patient isolation and contact precautions during the hospital stay. After validation, the score was shown to accurately identify patients with persistent VRE colonization upon admission, representing a suitable option as (i) a complementary method yielding preliminary results significantly more quickly than culture-based VRE detection techniques and (ii) an alternative strategy for VRE detection in settings in which microbiological VRE screening is not routinely performed due to limited resources.

## INTRODUCTION

Vancomycin-resistant enterococci (VRE) are pathogens with an increasing prevalence worldwide, thus posing a significant challenge for health care systems (1–3). Patients colonized with VRE develop VRE infections more frequently and additionally serve as potential sources of nosocomial VRE transmission to other patients and their environment (4–6). To prevent the spread of VRE in the hospital setting, extensive infection control measures are usually implemented (7, 8). In addition to microbiological screening, disinfection of contaminated surfaces, and implementation of antimicrobial stewardship (AMS) programs, strategies for control of VRE spread widely rely on contact precautions, i.e., isolation of colonized patients (9, 10). Patient isolation has been shown to compromise clinical care and pose a significant organizational and financial challenge (11).

Once a patient is VRE colonized, this state is assumed to persist permanently or for a long period of time (12–14). Hence, in settings adopting infection control measures against VRE, affected patients are often “labeled” as VRE carriers, resulting in the preventive application of contact precautions upon hospital admission, which are discontinued only after colonization has been ruled out by means of negative cultures (7). The implementation of contact precautions requires significant logistic, human, and financial resources. Based on data for methicillin-resistant Staphylococcus aureus (MRSA) obtained from a German surgical ward, the direct and indirect costs for contact precautions amount to 371.95 euros per patient hospital-day (11). Similarly, increased implementation of these measures has been shown to correlate with inversely proportional compliance rates among health care workers (15). Thus, effective strategies for reassessment of the colonization status of patients with a history of VRE are key for a rational implementation of contact precautions and an adequate allocation of financial and human resources.

In a recent study, we observed that a considerable number of patients experience a spontaneous VRE clearance over the course of time, especially if risk factors associated with relapse or long-term colonization, e.g., antibiotic treatment or prolonged hospital stay, are absent (16). Consequently, the value of risk stratification for prediction of VRE colonization status upon admission should be considered a complement to microbiological testing. This would allow for a patient-adapted implementation of contact precautions in order to effectively prevent VRE transmission while avoiding the unnecessary isolation of patients that have potentially cleared colonization. In addition, a clinical, risk-based strategy of colonization assessment would represent a suitable alternative for health care facilities that do not perform routine screening of patients with history of VRE upon readmission due to logistic or financial constraints.

We therefore developed a stratification score (the *pre*dictive *v*ancomycin-resistant *ent*erococci [PREVENT] score) based on clinical risk factors to predict the likelihood of VRE colonization upon admission for patients with a history of VRE, thus enabling timely and rational implementation of infection control measures before the results of microbiological testing and current VRE carrier status are available.

## RESULTS

**FIG 1:**
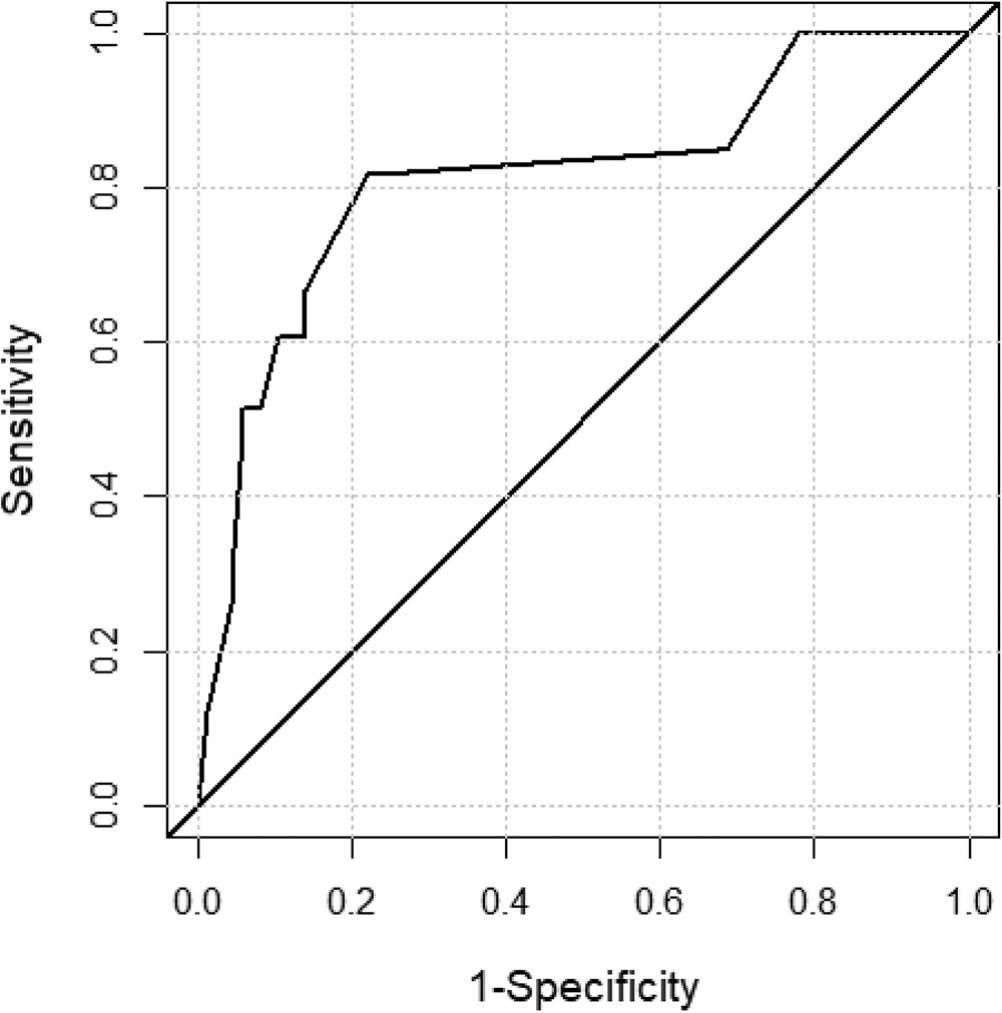
ROC curve of the logistic regression model.

**FIG 2:**
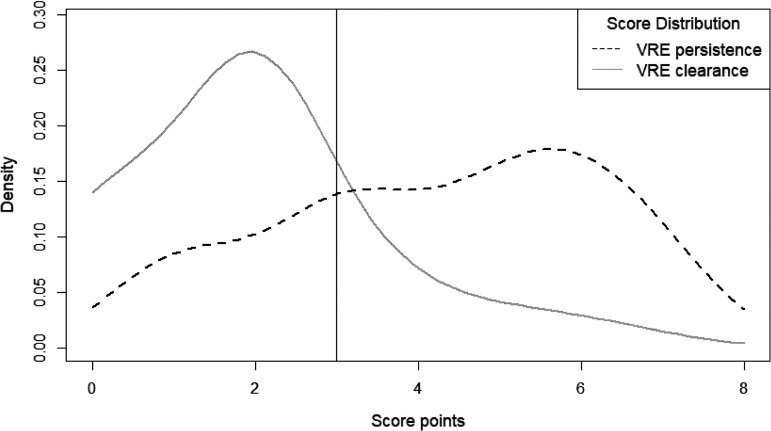
Distribution of score points in both groups in the validation cohort. The density of score points in patients with VRE persistence (black dashed line) versus VRE clearance (gray solid line) is shown. A cutoff value of 3 is indicated by the vertical black line.

**FIG 3:**
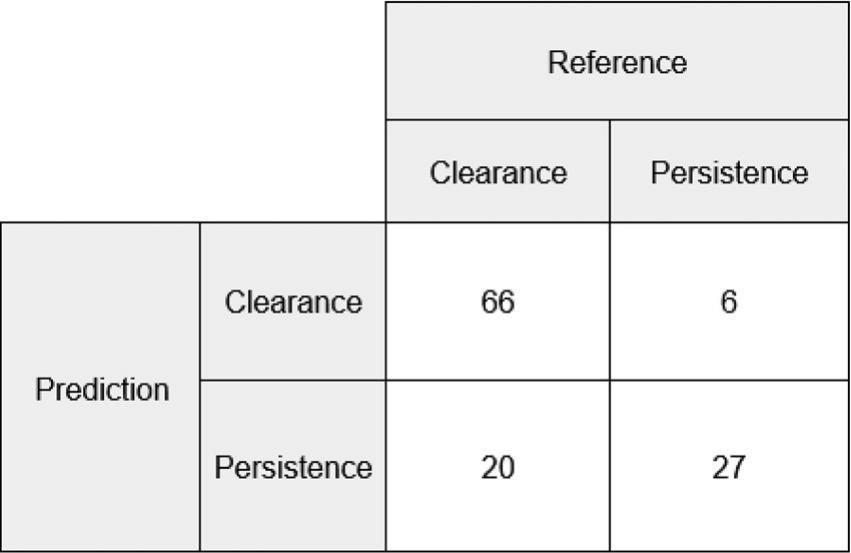
Classification of the PREVENT score. The observed classification is shown in the columns, and the predicted classification is shown in the rows.

The training cohort comprised 79% (*n* = 448) and the validation cohort 21% (*n* = 119) of all cases. The training and validation cohorts are comparable regarding the sex proportion (41% versus 40% female) and median age (58 versus 62 years). The results of the univariable analysis (absolute numbers, proportions, and *P* values) are displayed in Table 1. The variance inflation factor did not indicate the presence of multicollinearity. After the inclusion of all risk factors with a *P* value of <0.20 into the logistic regression model and backward selection, age of ≥60 years, present hemato-oncological disease, cumulative systemic intravenous or oral antibiotic treatment for >4 weeks, and VRE infection were identified as independent risk factors for VRE persistence (Table 2) and were included in the PREVENT score. The Hosmer and Lemeshow goodness-of-fit test resulted in a rejection of the null hypothesis, *P* = 0.225 indicating a good match of the observed and predicted risk of the training data. The receiver operating characteristic (ROC) and area under the curve (AUC) of 0.81 (95% confidence interval [CI], 0.72 to 0.91) indicate acceptable discrimination (Fig. 1). The score for each risk factor can be found in Table 3. The maximum score in the validation cohort was 8 points. Patients with VRE clearance and VRE persistence had a median score of 2 (interquartile range [IQR] = 1) and 5 (IQR = 3), respectively. Fig. 2 displays the density of score points of both groups in the validation cohort. A score of 3 points was defined as the cutoff value. Overall, the PREVENT score predicted the persistence of VRE colonization at readmission in the validation cohort with a sensitivity of 82% (95% CI, 65 to 93%), a specificity of 77% (95% CI, 66 to 85%), a positive predictive value of 57% (95% CI, 42 to 72%), and a negative predictive value of 92% (95% CI, 83 to 97%). In total, 78% of all patients were correctly classified. Fig. 3 displays the observed classification into the two groups versus the prediction using the PREVENT score.

**TABLE 1:** Univariable risk factor analysis

Risk factor	Total	VRE persistence	VRE clearance	*P*
	*N* (%)	*N* (%)	*N* (%)	
Age ≥60 yrs	232 (51.8)	114 (59.4)	118 (46.1)	0.01
Sex (female)	182 (40.6)	81 (42.2)	101 (39.5)	0.56
Hemato-oncological disease	231 (51.6)	113 (58.9)	118 (46.1)	0.01
Liver insufficiency	81 (18.1)	38 (19.8)	43 (16.8)	0.42
Renal insufficiency	194 (43.3)	81 (42.2)	113 (44.1)	0.68
Immunosuppressive disease	299 (66.7)	134 (69.8)	165 (64.5)	0.24
Antibiotic treatment for >4 wks	274 (61.2)	153 (79.7)	121 (47.3)	<0.001
VRE infection	83 (19.5)	50 (26.0)	33 (12.9)	<0.001
Previous antibiotic treatment	398 (88.8)	181 (94.3)	217 (84.8)	0.002
Hemodialysis	70 (15.6)	35 (18.2)	35 (13.7)	0.19
Admission from other hospital	110 (24.6)	54 (28.1)	56 (21.9)	0.13
More than 1 hospital admission	271 (60.5)	126 (65.6)	145 (56.6)	0.05

**TABLE 2:** Final multivariable model

Predictor	Regression coefficient (95% CI)	*P*
Age ≥60 years (yes/no)	0.82 (0.40–1.25)	<0.001
Hemato-oncological disease (yes/no)	0.43 (0.01–0.85)	0.046
Antibiotic treatment >4 weeks (yes/no)	1.50 (1.04–1.95)	<0.001
VRE infection (yes/no)	0.72 (0.19–1.25)	0.007

**TABLE 3:** PREVENT scoring

Predictor	Score
Age ≥60 yrs	
Yes	2
No	0
Hemato-oncological disease	
Yes	1
No	0
Antibiotic treatment for >4 wks in the previous 12 mo	
Yes	3
No	0
VRE infection	
Yes	2
No	0

## DISCUSSION

VRE increasingly pose a challenge for health care systems worldwide, predominantly affecting vulnerable patients with risk factors for colonization and infection. Since decolonization strategies as established, e.g., for MRSA (8, 17) have been lacking until now for the active elimination of VRE from the gastrointestinal tract (10, 18), strategies to prevent nosocomial transmissions have relied on hand hygiene, environmental disinfection, AMS programs, and contact precautions (7, 8, 17). The latter constitute a reliable method of breaking transmission chains. However, contact precautions have been described as potentially leading to unintended adverse consequences for patients, mainly regarding their psychological well-being and safety (19, 20). Hence, tools for rapid assessment of VRE colonization status that allow the timely and rational implementation of contact precautions are necessary. In this study, we aimed to develop a clinical prediction score system for VRE carriage to overcome the major problem of the unnecessary isolation of patients with a history of VRE colonization who might have experienced a (spontaneous) clearance prior to (re)admission.

When applying the developed score, we were able to reduce the need for isolation from 100% to 39% by only missing 18% of those with VRE colonization. Thus, both adverse effects on patients due to intensified contact precautions and harmful economic factors can be reduced to a minimum, while preventing nosocomial VRE spread. In total, 23% of patients with an observed loss of VRE carrier status were misidentified as VRE carriers by the score. Therefore, we recommend to further enhance the accuracy of the predictive score system by performing a routine microbiological screening upon readmission.

Clinical models for the prediction of colonization with multidrug-resistant organisms have been described, rendering mixed results (21–23). With respect to VRE, previous score systems have focused on predicting colonization upon admission to general hospital wards or intensive care units (24, 25). Low prevalence values of VRE colonized patients in these studies resulted in low positive predictive values. For example, one of these clinical scores displays a positive predictive value of 15.2% (25), which implies a false-positive proportion of approximately 85%. Consequently, the usability of this tool would be limited in the clinical setting, as many patients would be erroneously isolated. With higher prevalence values among patients with a history of VRE colonization, we achieved a higher positive predictive value (57%) with our score, resulting in a better applicability in the clinical setting.

Our study has some limitations. First, the training and validation cohorts comprised only patients from the same hospital displaying similar epidemiological and clinical characteristics, therefore limiting the generalization of the results. To guarantee the validity of the score, future investigations warrant external validation in an independent population. Second, information on the transitory risk factors acquired prior to readmission, e.g., in the context of treatment in other hospitals or clinics, might be incomplete for some patients, resulting in an information bias. Third, the prediction period took place before the coronavirus disease 2019 (COVID-19) pandemic, whereas the score validation was performed during the pandemic. Thus, organizational differences might have affected the present results. However, the developed model does not directly rely on contextual factors and is based solely on patient-related risk factors. Furthermore, the score’s development and performance rely on the sensitivity of the gold standard diagnostic method. Culture-based methods of VRE detection display a limited sensitivity. However, this is a hurdle for routine microbiological procedures in general, resulting in limitations intrinsic to clinical score designs.

In summary, we developed a risk factor-based score to predict the persistence of VRE colonization upon admission. The PREVENT score is a straightforward tool to identify patients with a high likelihood of VRE persistence and to avoid unnecessary infection control measures.

## MATERIALS AND METHODS

### Ethics.

All strategies and investigations were part of routine surveillance and infection control activities carried out in accordance with the national recommendations for VRE of the Robert-Koch Institute, Germany. All data were anonymized and cumulatively analyzed so as to avoid patient traceability. No additional patient data were collected for the purpose of this investigation. Formal consent was therefore not required.

### Setting.

The study was conducted at the 1,527-bed University Hospital Münster (UKM), a tertiary care center with approximately 65,000 patient admissions every year. The hospital regulations stipulate a screening for VRE for all high-risk patients (i.e., hemato-oncological patients) or patients with a history of VRE colonization or infection (microbiological detection of VRE from the infected site plus signs of infection). In accordance with national guidelines (10), extensive hygiene precautions are applied for all VRE-positive patients, including contact precautions, hand hygiene, and daily disinfection of the patient environment. All persons who enter the patients’ rooms, e.g., medical staff or visitors, wear personal protective equipment (single-use gloves and gowns). Hygiene measures are discontinued when three consecutive anorectal swabs, with an interval of at least 1 week between every swab, are negative. In case of (re)admission, the last negative swab must not be older than 4 weeks.

### Study design.

Over a 2-year period (October 2016 to October 2018), all patients (inpatients and outpatients) with a positive rectal screening for VRE colonization were included in the training data set to build the predictive model. VRE colonization was defined as VRE persistence if patients with a history of VRE colonization were still colonized at readmission. Accordingly, VRE clearance was assumed if no colonization could be detected at readmission in these patients. Potential risk factors for a persistent VRE colonization at readmission were documented. Documented potential risk factors were published in a previous study (16). Furthermore, the date and result of rectal swabs for VRE testing were documented. A second cohort was determined in a succeeding 11-month period (February to December 2020) for the validation data set of the final model and the predictive score.

### Statistical analysis.

Univariable analysis for categorical variables was performed using the chi-square test. All predictors with a *P* value of <0.20 were included into the logistic regression model. Testing for multicollinearity was performed using the variance inflation factor. Stepwise backward selection was performed for variable selection. The goodness of fit of the resulting model was evaluated using the Hosmer and Lemeshow goodness-of-fit test and receiver operator characteristic (ROC)/area under the curve (AUC). Score points were defined based on the coefficients of the predictors in the logistic regression model. The risk factor with the lowest coefficient was given one point for an increase of one level. Proportional to this, score points were defined for all other independent risk factors. The score was calculated for all patients in the training and validation data sets. The sensitivity, specificity, positive predictive value, negative predictive value, and balanced accuracy were calculated for the spectrum of score points. The cutoff value for classification into the clearance group or persistence group was set for a score point value where a sensitivity of 80% in the training cohort was reached. All statistical analysis was performed using R Studio version 1.3.1056 (R version 3.6.3) (The R Foundation, Vienna, Austria).
